# A Shot in the Arm for AIDS Vaccine Research

**DOI:** 10.1371/journal.pmed.0020036

**Published:** 2005-01-18

**Authors:** David D Ho

## Abstract

Why haven't we developed an HIV vaccine yet? And will the new roadmap from the Global HIV/AIDS Vaccine Enterprise help our efforts towards vaccine development? Ho addresses these crucial questions

The scientific strategic plan of the Global HIV/AIDS Vaccine Enterprise, published in this month's *PLoS Medicine*, is a clear and cogent document describing how major funders and stakeholders in HIV vaccine development should move forward in a collaborative fashion [1]. There is no doubt that this roadmap will be regarded as a useful instrument to bring greater cohesion and coordination to the field. The individuals who championed this effort should be commended for providing a great service to the scientific community. It is an excellent start to a continuing dialogue of utmost importance.

## The Challenge

Why is it that we still do not have a protective vaccine against HIV 22 years after its initial identification? Many possible explanations come to mind.

In the natural course of HIV infection, the virus wins 99% of the time, showing that specific immunity in an infected person is unable to completely clear the virus. We have also known for over a decade that primary HIV isolates are relatively resistant to antibody neutralization, probably because of a “protective shield” on the viral envelope glycoproteins, consisting of variable loop sequences and extensive N-linked glycosylations. Another explanation is the extreme plasticity of HIV that allows new viral variants to evade immune recognition in the same way that they escape from drugs. Moreover, superinfection by a second viral strain has been documented in a number of individuals who have already mounted immune responses to the initial HIV infection. Yet another problem is that the AIDS research community has yet to uncover the correlates of immune protection in vivo. Lastly, proven vaccine approaches from the past have either failed (whole killed virus and subunit vaccines) or faced seemingly insurmountable regulatory hurdles (live attenuated virus vaccine).

**Figure 1:**
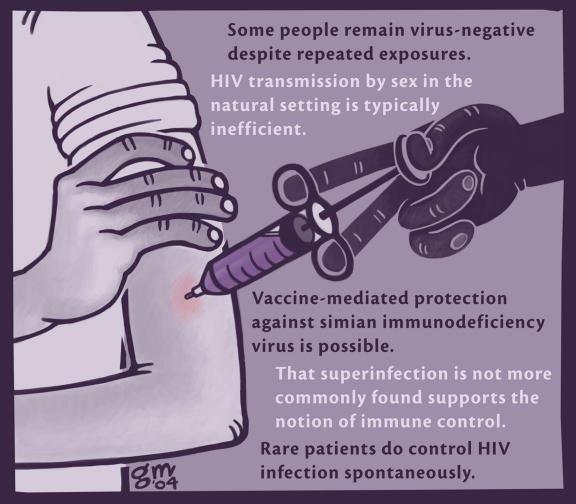
Rays of Hope: Clinical and Experimental Observations Suggesting That an HIV Vaccine Is Feasible (Illustration: Giovanni Maki)

Given these daunting obstacles, why have so many continued in the long struggle to develop an HIV vaccine? The answer must lie, in part, in the noble cause at hand. Yet there are also some encouraging clinical and experimental observations (Figure 1). Rare patients do control HIV infection spontaneously. Certain people remain virus-negative despite repeated exposures. That superinfection is not more commonly found supports the notion of immune control. Vaccine-mediated protection against simian immunodeficiency virus is indeed possible using live viruses attenuated by specific mutations or by pharmacological interventions. Finally, and perhaps most importantly, HIV transmission by sex in the natural setting is typically inefficient (and thus easier to block), unlike most experimental challenge systems employed in monkey studies to date. Collectively, these findings provide a ray of hope to push on.

## The Enterprise

The scientific strategic plan of the Enterprise is spot-on in identifying the major roadblocks in HIV vaccine development, as well as in establishing the key scientific priorities as we see them today [1]. It rightly recommends the formation of a growing alliance of organizations to foster a better collaborative spirit that could lead to, among other things, stronger political support and increased funding. The proposed greater coordination and management, sharing of information, technologies, and reagents, and harmonization of standards, assays, and approaches could only add to our overall efforts.

One might ask, however, whether there are potential downsides to the plan. In the name of continuing this important dialogue, I would like to offer one general concern. Arguably, the reason for the lack of an effective HIV vaccine today is rooted in the basic problems posed by the virus itself. What we need foremost are new scientific solutions, although a prim and proper “process and structure” in our approach will be helpful. The needed breakthroughs to develop a vaccine will likely emerge from the creativity of scientists doing fundamental research that is free of preconceived biases. It is my contention that great new ideas are as likely to come from curiosity-driven basic studies as from the mission-oriented approach that is represented by the new proposal. Therefore, the leadership of the Enterprise must safeguard against the kind of “group think” that is so pervasive in large collaborative endeavors of this nature. The views of a small number of researchers, no matter how smart or accomplished, must not supersede the collective wisdom of the scientific community at large.

No doubt important contributions will be made by scientists working outside of the Enterprise. Measures should be taken to ensure that their views and approaches, even if deemed unconventional, are not stifled by the newly established system. Likewise, their research support should not be compromised because the creation of the Enterprise concentrates the funding into the hands of a relatively small number of designated scientists. To me this is a serious risk given the current “flat funding” at the National Institutes of Health.

## The Future

The authors of the “The Global HIV/AIDS Vaccine Enterprise: Scientific Strategic Plan” have laid out a timely and insightful plan to address perhaps the greatest public-health need of the millennium. This document and its later revisions will serve as useful guideposts for the AIDS vaccine development effort for years to come. To be successful in this mission, our research community will ultimately need a specific “scientific blueprint” for making an HIV vaccine. That day will come only after we get another shot in the arm, infusing us with new knowledge and know-how. Is there any doubt that we need to redouble our investment in basic research on the challenges posed by HIV?
