# Correction: A Shot in the Arm for AIDS Vaccine Research

**DOI:** 10.1371/journal.pmed.0020299

**Published:** 2005-08-30

**Authors:** David D Ho

In *PLoS Medicine*, vol 2, issue 2, DOI: 10.1371/journal.pmed.0020036

David D. Ho states that he should have declared as a competing interest that two members of his research team and Ho himself are co-inventors on two candidate vaccines that are in clinical development. For this effort, they also receive funding from the International AIDS Vaccine Initiative.

